# A Distributed Real-Time Transcoding CDN System Design and Performance Simulation Study

**DOI:** 10.3390/s22051945

**Published:** 2022-03-02

**Authors:** Yuan Ren, Cheng Huang, Yongbo Lv, Wanjun Lv, Han Zhang

**Affiliations:** School of Traffic and Transportation, Beijing Jiaotong University, Beijing 100044, China; 7309@bjtu.edu.cn (Y.R.); 12114200@bjtu.edu.cn (C.H.); 18114022@bjtu.edu.cn (W.L.); 19114030@bjtu.edu.cn (H.Z.)

**Keywords:** distributed real-time transcoding, CDN systems, resource negotiation, resource storage policies

## Abstract

Content Delivery Network (CDN) technology is one of the core technologies for performance optimization of mobile WEB applications, but it often encounters bottlenecks in video storage. In this paper, based on studying the basic technical model of distributed real-time transcoding CDN, we propose an overall technical architecture of a distributed real-time transcoding CDN system, present the business process of distributed real-time transcoding CDN resource negotiation in the model, design a resource storage policy model and algorithm, including the hot storage ratio resource storage value evaluation method and storage resource allocation algorithm. Through simulation experiments, we verify the technical efficiency of the distributed real-time transcoding CDN system by simulation experiments.

## 1. Introduction

CDN is one of the core technologies for performance optimization of mobile WEB applications. It achieves increased transmission speed by moving static resources to caching servers close to user requests [1]. It also reduces the load on the backbone communication network by reducing the non-essential occupation of backbone communication resources by such static resources. The resources suitable for CDN transmission usually include static video, audio, image, and web page. Among them, video resources occupy the most storage space. On the one hand, video resources consume more storage space than other resources storage itself; on the other hand, the same video content often exists in several different video formats, and CDN servers need to store the same video content in different video formats to meet users’ demands, which further aggravates the occupation of storage space. The main reasons for multiple formats of video content include many kinds of formats of playback traffic, different playback formats supported by the software, and various screen resolutions of users’ mobile terminals. This phenomenon is more prominent in mobile WEB applications. According to the survey, the same video content is often stored in more than 120 different versions. Although many different versions of video can meet the different needs of different users, it also greatly increases the storage pressure of CDNs [2,3,4,5].

Recently, there have been many CDN-related researches, such as the optimization of DNS for CDNs, the combination of P2P and CDNs, and large-scale video CDN technology. The EUE principle from the view of resource provision and the peer resource request mechanism was presented in [6]. A real-world hybrid CDN–P2P system was built for live streaming, called LiveSky, which helped bridge the gap between CDN and P2P in [7]. The paper [8] proposed one novel Web Services standard-supported CDN–P2P loosely-coupled hybrid and management model. In order to offload the original CDN server, cross-region content replication was conducted collaboratively in [9]. A novel methodology to detect the statistical differences in the performance for CDNs and large-scale distributed systems were built in [10]. Paper [11] discussed the ETPC algorithm for the efficient transmission of the filtered real-time heart-rate activity data. Paper [12] built a new framework based on Cloud Content Delivery Networks (CCDNs) to capture the dynamic characteristics and quantifies the node transmission efficiency. Malektaji et al. proposed a deep reinforcement learning (DRL) content migration technique for a hierarchical edge-based CDN [13].

CDN technology is important for improving the transmission speed of mobile WEB, but the bottleneck problem of CDN technology in terms of storage space has not been addressed in a large number of existing studies on CDN technology. In order to solve this problem, this paper proposes distributed real-time transcoding CDN technology optimization, which tries to solve the problem of excessive storage space consumption of CDN servers storing multiple formats of videos of the same content, so as to improve the processing capability of mobile WEB systems for multi-format same-content resources and shorten the response time of users to obtain the resources. The goal of this research is to enable significant improvements in the processing power of multi-format same-content resources. A performance improvement of more than 30% can be achieved when there are more formats of the same content.

The main contributions of this study are as follows.

This paper examines the key technology and algorithm in the distributed real-time transcoding CDN control module, namely the storage policy. Two key elements are required to study the storage strategy.

- The evaluation of the storage value of each resource. Evaluating the storage value of a resource enables a comparison among the resources and lists the storage solution with the most storage value.
- The storage allocation algorithm, which determines how storage space should be allocated based on the storage.

In the following paper, Section 2 designs the distributed real-time transcoding CDN system. Section 3 elaborates on the resource storage policy models and algorithm in detail. The simulation results and relevant discussion are presented in Section 4. Finally, Section 5 concludes this paper.

## 2. Distributed Real-Time Transcoding CDN System Design

Traditional CDNs do not have an effective solution when facing the problem of storing a large number of videos with different formats of the same content. By introducing the distributed real-time transcoding method, this problem can be effectively solved. Therefore, by adding real-time transcoding devices to the CDN server and increasing the CDN real-time transcoding function, the number of videos of the same content stored by the CDN can be reduced and the total number of videos of different contents stored by the CDN server can be increased [14,15]. Compared to traditional CDNs, this method has a better hit rate and improves the user’s experience. However, the architecture needs to be studied on top of the traditional CDNs, adding a real-time transcoding module and a system control module.

### 2.1. Overall Architecture Design of Distributed Real-Time Transcoding CDN System

In order to maximize the total user satisfaction, the distributed real-time transcoding CDN system adds two core functions to the traditional CDN system: the real-time transcoding module and the system control module. Among them, the real-time transcoding module needs to design a set of hardware that can meet the real-time transcoding of CDN application scenarios, and at the same time develop corresponding software interfaces, so that the transcoding decision module can call these interfaces to realize real-time transcoding. GPUs, FPGAs, and dedicated chips have their own advantages in meeting the basic requirements. They can all be used in CDN real-time transcoding scenarios. The system control module needs to design a set of control systems that can control the real-time transcoding hardware to complete the functions of the distributed real-time transcoding CDN system, and the main functions include: resource heat analysis, CDN storage strategy, resource negotiation, and device status acquisition. In order to achieve the above functions, communication protocols and corresponding business processes are also needed to cooperate between the network elements.

**Figure 1:**
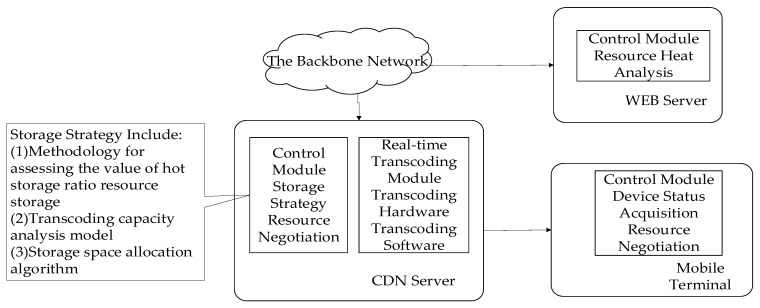
Architecture of the distributed real-time transcoding CDNs.

The overall structural design is shown in Figure 1.

As shown in Figure 1, the two functional modules are distributed on various network nodes (WEB server, CDN server, and mobile terminal). The real-time transcoding module is placed on the CDN server, while the control module requires the joint participation of the client, CDN server, and WEB server. The control module requires the WEB server to be responsible for the calculation of resource hotness and provide the query interface of resource hotness, and the client and CDN server need to cooperate to complete the negotiation process, while the CDN server completes the resource storage policy function.

The WEB server is the core server of the website, storing all the raw video data with comprehensive information, and is more suitable for evaluating the heat of the resources. Since it is in the central node, it tends to have a stronger CPU processing power and more storage space, but it is often farther away from the user, has a longer communication latency with the user, less bandwidth, and is also highly likely to lose packets due to dry node router congestion.

The distributed real-time transcoding CDN server has the full functionality of a CDN server and extends the functionality of the CDN server by adding real-time transcoding devices to the generic server through a generic interface. The CDN server needs to perform optimization processing between storing multiple video formats and hardware real-time conversion, and cannot perform distributed real-time transcoding for all the videos. The CDN server is responsible for scheduling its own resources to complete the optimization process, but also to give users a better experience while controlling its own energy consumption. Among its functions, storage policy and resource negotiation are particularly important and represent a research gap.

CDNs are accessed via the WEB and use the HTTP protocol for data transfer, which is inherently very scalable and can be used to transfer interactive information from distributed real-time transcoding CDN systems. This method is versatile and can be used with various HTTP proxies or reverse proxies. If the HTTP standards were not used, such as adding the relevant interaction information after a TCP message and then using the TCP message to communicate, it would need the system to support multiple application layer communication protocols.

### 2.2. Business Processes for CDN Resource Negotiation

**Figure 2:**
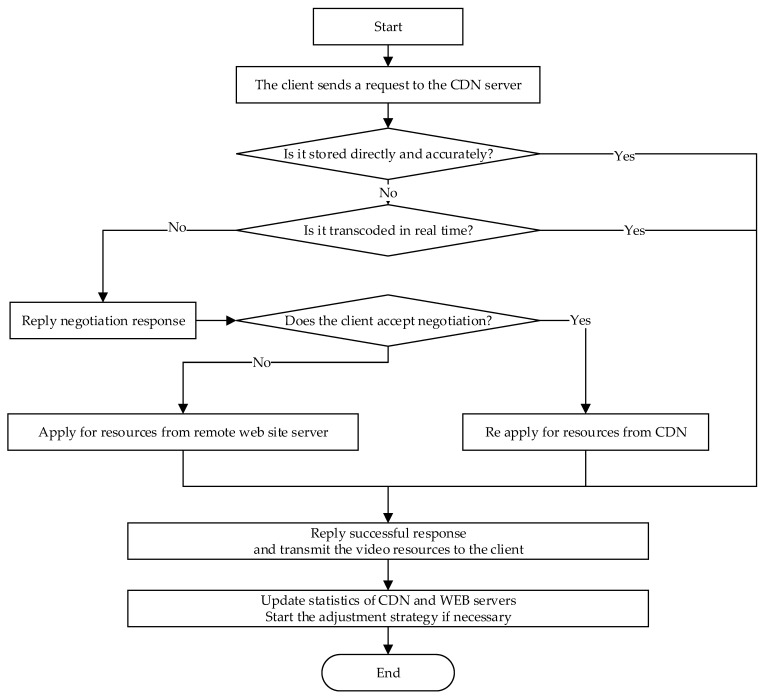
Flow-process diagram of the data service.

The business process is mainly used to describe the dynamic operation process of the whole distributed real-time transcoding CDN system. This business process is mainly composed of control negotiation, resource preparation, resource transfer, resource hotness statistics, real-time transcoding load statistics, and storage resource adjustment. The first step of the business process is control negotiation, and then resource preparation (including CDN direct reading, CDN real-time transcoding, and WEB server remote acquisition) is executed after the control negotiation is completed, after which the resources are transferred to the client, and finally WEB server resource heat statistics, CDN resource load statistics, and storage resources in the CDN server are adjusted regularly. The business process is shown in Figure 2.

The business process is divided into the following steps:

1. To increase the probability of successful first-time negotiation, the client directly sends an HTTP message with the URL of the video resource and details of the video format he/she wishes to obtain.
2. Return the video resource if the CDN server can meet the other party’s demands.
3. If it does not work to trigger the negotiation process, the CDN server lists the details of the videos it can provide.
4. During the negotiation process, the CDN server provides detailed information about the resources, in addition to the server’s expected latency and download speed, and the client can choose the file format he/she needs, including the several acceptable formats based on this information.
5. If the negotiation fails, the CDN server obtains the video resources from the remote WEB server.
6. After completing the client request, the access record is counted, and the information, such as hotness and the real-time transcoding load, is counted. Whether the CDN retains the data depends on the result of the statistics, and the CDN has the right to decide whether to retain the data sent from the remote end.

**Figure 3:**
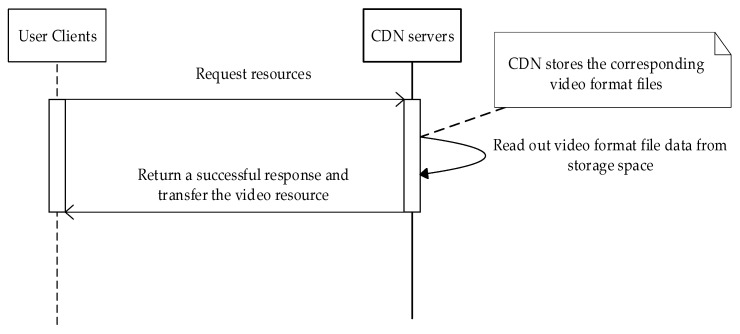
Sequence diagrams of CDN direct storage of video format.

**Figure 4:**
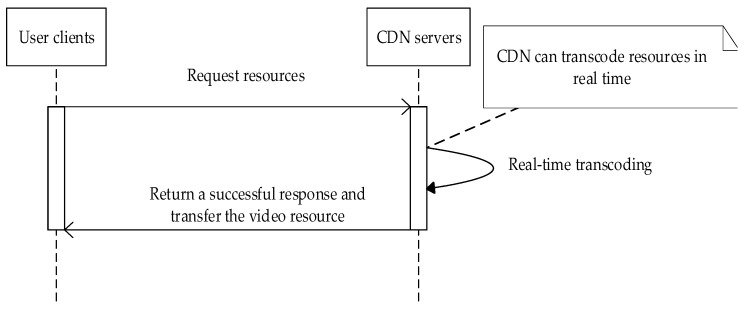
Sequence diagram of CDN feasible transcoding.

To clearly illustrate how the various network elements are involved, several timing diagrams of the processing logic are presented in Figure 3 and Figure 4.

As shown in Figure 4, if the CDN stores a feasible transcoded video format, the CDN can generate a video format file through real-time transcoding and pass the data to the user client after the client sends a request.

## 3. Resource Storage Policy Models and Algorithm

Resource storage policy is the key technique and algorithm in the control module of distributed real-time transcoding CDN systems. Three key elements are needed to study the storage strategy: (1) an assessment of the storage value of each resource—assessing the storage value of a resource enables a comparison among the resources and lists the storage solutions with the most storage value. (2) Transcoding capacity analysis—real-time transcoding technology is constrained by the transcoding capacity, so transcoding capacity analysis is needed. (3) Storage space allocation algorithm—the storage allocation algorithm is based on the storage value and transcoding capacity analysis to decide how to allocate the storage space.

### 3.1. Methodology for Assessing the Value of the Hot Storage Ratio Resource Storage

A video resource can be counted by the WEB server for the number of times it is requested by users, and this is used to calculate how this video resource is likely to be accessed in future periods. It is reasonable to measure the storage value of a resource by how much it is used, and when it is heavily used, it has a higher storage value. However, using only the hotness of a video resource for the measured storage value would ignore the size of the storage space occupied by each video resource, so the hotness needs to be corrected with the storage space size. To measure the storage value, the hot storage ratio is defined here:

(1) $$hsr=\left(h\times t\right)/s$$

where *hsr* is the hot storage ratio of a video resource, *h* is the hotness of the video resource, *t* is the duration of the video content, and *s* is the storage space occupied by the video resource.

In the application domain of real-time transcoding, the sum of the hot storage ratios of all its feasible transcodes (including the original format) should be used to assess the storage value of this resource, which is defined here as the transcoding hot storage ratio.

(2) $$hsrt=\sum_{vf\in VF}hsr_{vf}$$

where *hsrt* is this video transcoding hot storage ratio, *vf* is a video format, *VF* is the set of video formats that the video format can feasibly transcode, and *hsrvf* is the *vf* video format hot storage ratio.

If all video resources stored by a CDN server correspond to unique content, then the overall transcoding hot storage ratio of the CDN server is equal to the sum of the real-time transcoding hot storage ratios of all video resources.

(3) $$hsrtt=\sum_{vf\in \mathrm{VFCDN}}hsrt_{vf}$$

where *hsrtt* is the overall real-time transcoding hot storage ratio, *vf* is a video format, VFCDN is the collection of all video formats stored by the CDN server, and $hsrt_{vf}$ is the real-time transcoding hot storage ratio of each video resource.

To reduce the burden on the real-time transcoding module or to save energy consumption, the CDN server needs to store multiple format versions of the same video content, and when multiple video formats corresponding to the same video content are stored in the CDN server, the overall real-time transcoding hot storage ratio is not the sum of the real-time transcoding hot storage ratios of all the video resources, but needs to subtract the part of the same content video resource that is double counted.

(4) $$hsrtt=\sum_{vf_{0}\in \mathrm{VFCDN}}hsrt_{vf_{0}}-\sum_{vf_{1}\in VFD}hsr_{vf_{1}}$$

where *VFD* is the set of video formats stored in the CDN server that can be converted to each other resulting in double counting, and *hsr_vf_*_1_ is the hot storage ratio of the video formats being double counted.

When the dynamic adjustment of the CDN’s storage resource allocation is required, a metric is needed to measure the amount of change in the value of the storage resources, namely the marginal hot storage ratio for this adjustment. The marginal hot storage ratio is the amount of change in the overall real-time transcoding hot storage ratio that would result from adding or subtracting another video format relative to the existing set of video formats.

(5) $$hsrm=hsrtt_{1}-hsrtt_{0}$$

where *hsrm* is the marginal hot storage ratio for this dynamic adjustment, $hsrtt_{1}$ is the overall transcoding hot storage ratio after the stored content adjustment, and $hsrtt_{0}$ is the overall transcoding hot storage ratio before the stored content adjustment. When the marginal hot storage ratio is positive, this adjustment leads to better user demand coverage; otherwise the opposite is true.

### 3.2. Storage Resource Allocation Algorithm

Based on the assessment of the storage value, the storage resources can be allocated by the corresponding algorithm. The storage allocation algorithm is performed on the basis of the hot storage ratio resource storage value assessment. If whether the stored video format, *vf*, is viewed as a 0–1 variable, where 0 means that the node does not cache the video and 1 means that the node stores the video, the total storage constraint is viewed as a knapsack sum. For example, the proposed model can be viewed as a class of knapsack problems. Since the backpack problem belongs to the NPC problem, solving the resource storage allocation model also belongs to the NPC problem, which is a difficult problem to solve. Moreover, for the distributed real-time transcoding CDN system, the resource hotness state keeps changing due to user requests, which further aggravates the difficulty of solving the problem, even if the optimal solution is found in the allowed time. For example, regarding the storage content that should be in the CDN at each moment, the resources cannot be quickly updated to each CDN, and the transmission of the video resources requires a large number of network transmissions and disk read/write operations to occur, resulting in network and CDN performance degradation. Therefore, from the practical application effect, using a heuristic strategy to dynamically perform resource updates is the least costly, feasible solution. Therefore, heuristic algorithms are designed for the resource storage allocation problem of distributed real-time transcoding CDN systems.

(1) Initial resource allocation algorithm.

In the initial resource allocation, the storage resource allocation list is obtained by using a greedy algorithm that includes only the video formats that generate the largest *hsrm* in the video content in the storage list. The initial resource allocation algorithm is actually optimal if the transcoding capacity is abundant, i.e., when the real-time transcoding capacity can satisfy all demands. This allocation can guarantee that the user receives the maximum satisfaction without considering the transcoding capacity constraint, and can guarantee that the subsequent dynamic adjustment does not need to obtain the video format of the existing video content from the WEB server again, when considering the transcoding capacity constraint. The pseudo-code is shown in Table 1.

Where v_list is a list of the video content, vf_v_map is a dictionary of video formats mapped to the video content, vf_hsr_map is a dictionary of video formats mapped to the hot storage ratios, and alloc_list is a list of output initialization storage resource allocations.

The algorithm steps are as follows:

1. Calculate the vf of the maximum hsrt for each video content.
2. Put the video format that has the largest hsrt into the container.
3. Sort the video formats in the container in hsrt descending order.
4. Remove the hsrt largest video formats from the container one-by-one, and place them into the CDN.
5. Perform this method until the CDN’s storage space is full.

The initialized resource allocation algorithm is allocated by the optimal allocation method that does not consider the transcoding capacity limit. Although the initialized allocation is not necessarily the optimal allocation, it can satisfy the vast majority of requests, and since the CDN stores the vf of the maximum hsrt, it only needs to keep the real-time transcoding data when it needs to be dynamically adjusted. CalcMaxHsrtVfOfV is to calculate the maximum Hsrt of the video content and return the resulting video format and its Hsrt. hsrt_max_map is a dictionary of the maximum Hsrt to the video content. sortVbyhsrt sorts all video content by Hsrt.

(2) Dynamic adjustment algorithm.

After initializing the storage allocation algorithm when the real-time transcoding capacity is insufficient, a dynamic adjustment method is used. The main consideration for dynamic adjustment is the real-time transcoding capacity. If the real-time transcoding capacity is saturated with usage, it is possible to increase the user request coverage by storing multiple copies of high-hot video content in a video format. If the real-time transcoding capacity is idle, it is necessary to reduce the number of the high-heat multiple copies of the video format. The pseudo-code is shown in Table 2.

Where alloc_list is the list of allocated storage resources for the input and output, GetMinHsrVfInCdn is the vf with the lowest hsr in the CDN, and the video content it corresponds to has only one copy of the video format in the CDN. getMaxHsrVfInMaxHsrtV is the video format for which the maximum hsr is solved, and this format is not in the CDN, but feasible transcodes exist for this format in other formats. CheckRTResource is to obtain the resource usage status, status is the resource usage status, overload means hardware conversion resource usage overload, and idle means that the hardware conversion usage status is idle. GetMinHsrVfInHotVf is to obtain the lowest Hsr hot video format. GetMaxHsrtVfNotInCdn is to obtain the maximum Hsrt video format that is not in the CDN.

The algorithm steps are as follows:

1. Check the status of real-time transcoding capabilities at regular intervals.
2. If the transcoding capacity is running at full load, free up disk space for the lowest hsrt video content and store multiple copies of the high heat video content in a video format.
3. If there is an idle real-time transcoding capacity, remove multiple copies of the high-heat video content from the video format and store more video content instead.

## 4. Simulation Experiments and the Performance Evaluation

In this section, the technical performance of the distributed real-time transcoding CDN system is verified through simulation experiments, and the results are evaluated and analyzed [16,17].

### 4.1. Design of the Simulation Experiments

(1) Principles of the simulation experiment design.

The simulation experiment uses discrete-event simulation software. The simulation of the application scenario is achieved by adding the module of the distributed real-time transcoding CDN system to the simulation software. The experiments are divided into three groups: one group uses distributed real-time transcoding CDN technology, another group uses normal CDN technology, and the last group does not use CDN technology and all the data is obtained from the WEB server. With these three groups of experiments, it is possible to clearly compare the effects.

Since the video format is an overwhelmingly large resource, the impact of latency on the quality of service is relatively small, and, in this paper, the focus is on the impact of the bandwidth. The bandwidth from the CDN server to the client is relatively abundant, while the CDN to the remote WEB server has bottlenecks or more uncertainties (for example, bandwidth jitters). These characteristics are simulated to highlight the network backbone node transmission bottlenecks, and the impact of latency is ignored in the present study, in order to reduce unnecessary simulation complexity.

(2) Treatment flow of the three sets of experiments.

The basic treatment of the three sets of experiments was as follows:

- Closed CDN group: this group of experiments is mainly used to compare the other two groups. It has a very simple business process, sending all client requests directly to the WEB server, which then sends the data to the client via the backbone network.
- Ordinary CDN group: this group of experiments is relatively more complex. There is a CDN in this network element, but here the CDN cannot be distributed via real-time transcoding. The CDN server only stores the video format resources that are larger than the hot storage; consider only the video format file hot storage ratio. After the client initiates a request, the CDN server receives the request, and if it stores the exact video format file, it returns it directly to the client; if not, it forwards the request to the WEB server, and the remote WEB server, after the backbone network, sends the data to the client.
- Distributed real-time transcoding CDN group: this group of experiments then exactly follows the method described in this paper for the relevant business processes.

(3) Simulation experiment parameters.

Networking parameters are parameters used to describe the state of the network, and they are fixed in the simulation experiments. The detailed parameters information is shown in Table 3.

Here, it is assumed that there are 3 CDN servers, each with 2 GB of CDN storage, and the users use WIFI access with a relatively stable network speed. To facilitate the validation of the effect and to simplify the complexity of the simulation run, the networking parameters in the present study are relatively small, and the actual situation may be several orders of magnitude higher than this. However, the simple networking model already speaks for itself, because when all these parameters are simultaneously increased by several orders of magnitude, neither does the CDN hit rate nor the user experience change. In addition to the networking parameters, a table of operational parameters is needed, which is presented in Table 4.

To simplify the simulation to highlight the focus of this study, only video services are used here. In order to fully validate the distributed real-time transcoding CDN system, simulation experiments are also needed in several different environments. The variable environmental parameters are presented in Table 5.

With the maximal video formats, the storage capacity of the CDN is at a stage where it is severely lacking and cannot store all formats of the video content.

(4) Indicators of the experimental results.

A common metric in CDN research is the CDN hit ratio; a CDN design with a high hit ratio inevitably reduces the overall latency for users, and this paper also uses the hit ratio metric. The hit ratio is defined as follows:

(6) hr=cdn_count/total_count

where hr is the CDN hit rate, *cdn_count* is the number of user requests answered by the CDN itself, and *total_count* is the number of all user requests.

The hit rate is the metric that directly affects the results, but it does not directly reflect the real-time user profiles, and the metric of the maximum number of real-time users needs to be added. Both metrics are uncertain and affected by the resources accessed by users and other random events, so the metric given here is the average of several simulation results.

### 4.2. Analysis of Results

**Figure 5:**
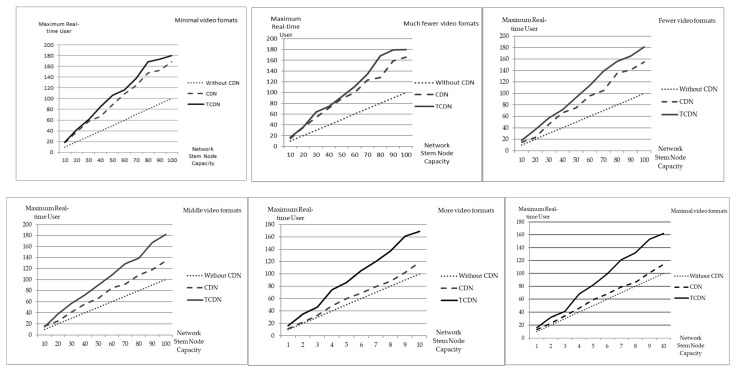
Diagram of the maximum real-time user supported in different video format environments.

The simulation results for the maximum number of real-time users in different video format environments are shown in Figure 5.

Among them, TCDN is a distributed real-time transcoding CDN technology, and the unit of network stem node capacity is MB.

As can be seen from Figure 5 and Table 6, the distributed real-time transcoding CDN and the regular CDN are significantly better than the case without a CDN. In the case where the number of videos is only 2 or 3, and the hot videos are very concentrated, the distributed real-time transcoding CDN can still support about 10–15% more users than the normal CDN. As the number of videos of the same content increases to 8, the distributed real-time transcoding CDN can support about 30% more users than the regular CDN. When the number of video formats is 13, the distributed real-time transcoding CDN can support about 42% more users than the regular CDN. When the number of video formats is 19, the distributed real-time transcoding CDN can support about 43% more users than the regular CDN.

Based on the results, it is clear that the number of supported users is bound to increase regardless of the environment, as long as the network trunk node is more capable. In the environment without a CDN, the size of the number of users depends only on the capacity of the network trunk nodes. When the number of videos of the same content is relatively small, the effect played by an ordinary CDN is much better, but still lower than the distributed real-time transcoding CDN. When the number of videos of the same content is relatively large, the effect played by an ordinary CDN is worse, or even closer to no CDN. When the number of each content video format increases, the capability of an ordinary CDN gradually diminishes, while the capability of the distributed real-time transcoding CDN decay is lower, and even no significant decay can be seen.

**Figure 6:**
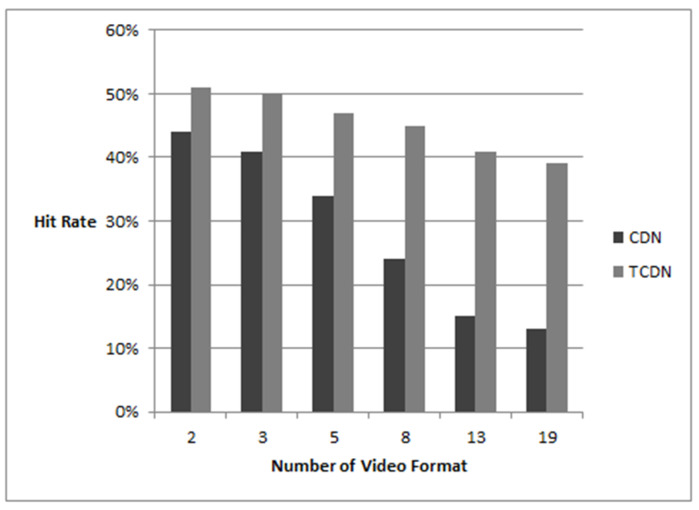
Hit rate column contrast chart.

As shown above, Figure 6 visually depicts the data in Table 7. The hit rate results for 6 kinds of simulation environments are shown in Figure 6. The no-CDN case does not have the concept of a hit rate, so no comparison is made. From the comparison graph, it can be seen that the hit rate of the normal CDN decreases rapidly with the number of video formats, while the distributed real-time transcoding CDN is much less affected. For 2 and 3 video formats, for the same content, the hit rate differs by 20%; for 5 video formats, the hit rate differs by 30%; and for 8 video formats, the hit rate differs by 46%. When the number of video formats is 13 and 19, the difference in the hit rate is 63% and 66%, respectively. The comparison method is that the difference between the TCDN and CDN is divided by the higher hit rate. When the number of video formats is greater than 5, there is a significant difference in the hit rate between them.

In summary, the distributed real-time transcoding CDNs can significantly improve the CDN performance of CDNs with multiple copies of the same content in video formats.

## 5. Conclusions

In this paper, we designed a distributed real-time transcoding CDN system with the goal of reducing the total user latency, presented the business process of distributed real-time transcoding CDN resource negotiation, and proposed a resource storage strategy, including a hot storage ratio resource storage value assessment method and a storage resource allocation algorithm, for the current mobile video resources that often exist in several different format versions, models, and algorithms. Finally, the practical value of this distributed real-time transcoding CDN system is verified by simulation experiments, which has obvious advantages over traditional CDNs. This paper mainly studies and analyzes video transcoding, but the distributed real-time transcoding CDN technology is also applicable to application scenarios, such as audio, images, compressed files, different encrypted format files, and different storage format documents.

The distributed real-time transcoding CDN has significantly improved the performance of CDNs, but it mainly faces the following two challenges: the distributed real-time transcoding CDN requires the system to update the original hardware, which increases some costs in the engineering field; simultaneously, in the environment with only one video format, the distributed real-time transcoding CDN system does not result in performance improvement.

Distributed real-time transcoding CDNs still have issues worth studying. Research on real-time transcoding technology is currently relatively easy to achieve through GPU transcoding, but FPGAs and special chips for converting multiple video formats to each other are technically difficult to use, but they have higher performance rates and better economic benefits than GPU transcoding; therefore, research on distributed real-time transcoding FPGAs and special transcoding chips should be strengthened to support distributed real-time transcoding CDN technology better.

## Figures and Tables

**Table 1:** Pseudo-code for initializing the storage resource allocation algorithms (overview).

Step	INIT_ALLOC (v_list, vf_v_map, vf_hsr_map, alloc_list)
1	FOR v in v_list
2	{vf, hsrt} = CalcMaxHsrtVfOfV (v, vf_v_map, vf_hsr_map)
3	hsrt_max_map[v] = {vf, hsrt}
4	vf_hsrt_list = SortVbyhsrt (hsrt_max_map)
5	i = 0
6	WHILE total_s + v_hsrt_list[i].hsrt < max_s
7	alloc_list.Add (v_hsrt_list[i].vf)
8	total_s += v_hsrt_list[i].hsrt

**Table 2:** Pseudo-code of dynamic adjustment algorithms (overview).

Steps	Dynamic_tuning (alloc_list)
1	WHILE true
2	Delay (wait_time)
3	status = CheckRTResource ()
4	IF status == overload
5	vfMinhsr = GetMinHsrVfInCdn ()
6	DelVf (alloc_list, vfMinhsr)
7	vfMaxhsr = GetMaxHsrVfInMaxHsrtV ()
8	StoreVf (alloc_list, vfMaxhsr)
9	IF status == idle
10	vfInHotVf = GetMinHsrVfInHotVf ()
11	DelVf (alloc_list, vfInHotVf)
12	vfNewV = GetMaxHsrtVfNotInCdn ()
13	StoreVf (alloc_list, vfNewV)

**Table 3:** Table of the simulated networking parameters.

Parameter Name	Parameter Value
Number of CDNs	3
CDN storage space	Close to 20 video files
User type	WIFI access (assuming a stable internet speed)

**Table 4:** Table of the simulated business parameters.

Parameter Name	Parameter Value
Maximum number of users	1000
Type of business	Access to video services
Video content distribution	Long tail distribution
Number of video contents	100
Real-time transcoding capability	Able to complete 80 percent of the total user requests

**Table 5:** Table of the environment parameters.

Environment Name	Number of Video Formats per Content
Minimal video formats	2
Much fewer formats	3
Fewer video formats	5
Middle video formats	8
More video formats	13
Maximal video formats	19

**Table 6:** Table of the maximum real-time user supported in different video format environments.

Network Trunk Node Capability	Minimal Video Formats			Much Fewer Video Formats			Fewer Video Formats		
	NO CDN	CDN	TCDN	NO CDN	CDN	TCDN	NO CDN	CDN	TCDN
10	10	18	19	10	15	17	10	15	18
20	20	38	42	20	35	36	20	23	37
30	30	58	61	30	54	64	30	46	57
40	40	67	85	40	71	74	40	66	72
50	50	89	106	50	89	92	50	75	93
60	60	109	116	60	100	111	60	96	114
70	70	124	138	70	123	134	70	105	139
80	80	148	168	80	128	168	80	135	156
90	90	153	173	90	159	179	90	141	165
100	100	168	180	100	166	180	100	155	181
**Network** **Trunk Node** **Capability**	**Middle Video Formats**			**More Video Formats**			**Maximal Video Formats**		
	**NO CDN**	**CDN**	**TCDN**	**NO CDN**	**CDN**	**TCDN**	**NO CDN**	**CDN**	**TCDN**
10	10	16	15	10	11	16	10	13	16
20	20	25	38	20	22	35	20	23	32
30	30	41	57	30	33	46	30	34	41
40	40	56	72	40	48	74	40	46	68
50	50	66	90	50	60	86	50	59	82
60	60	85	108	60	69	105	60	68	99
70	70	92	129	70	79	120	70	79	121
80	80	108	139	80	88	137	80	86	132
90	90	118	167	90	102	161	90	101	153
100	100	134	182	100	119	169	100	114	162

**Table 7:** Table of hit rates.

Number of Video Formats	CDN	TCDN
2	0.44	0.51
3	0.41	0.50
5	0.34	0.47
8	0.24	0.45
13	0.15	0.41
19	0.13	0.39

## Data Availability

The study did not report any data.
